# Introduction of *Lasiolepiota (Agaricaceae)*, a new genus with two new species from Laos and Thailand

**DOI:** 10.3897/mycokeys.142.201080

**Published:** 2026-10-30

**Authors:** Phongeun Sysouphanthong, Komsit Wisitrassameewong, Yuan S. Liu, Narumon Tangthirasunun, Else C. Vellinga, Naritsada Thongklang

**Affiliations:** 1 Center of Excellence in Fungal Research, Mae Fah Luang University, Chiang Rai, 57100, Thailand Center of Excellence in Fungal Research, Mae Fah Luang University Chiang Rai Thailand; 2 Biotechnology and Ecology Institute, Ministry of Agriculture and Forestry, P.O. Box 811, Vientiane Capital, Lao People's Democratic Republic Biotechnology and Ecology Institute, Ministry of Agriculture and Forestry Vientiane Capital Lao People's Democratic Republic; 3 Department of Biotechnology, Faculty of Science, Mahidol University, Bangkok 10400, Thailand Department of Biotechnology, Faculty of Science, Mahidol University Bangkok Thailand; 4 National Biobank of Thailand, National Science and Technology Development (NSTDA), Thailand Science Park, Khlong Neung, Khlong Luang, Pathum Thani 12120, Thailand National Biobank of Thailand, National Science and Technology Development (NSTDA), Thailand Science Park Pathum Thani Thailand; 5 Faculty of Agriculture, Forestry and Food Engineering, Yibin University, Yibin 644000, Sichuan, China Faculty of Agriculture, Forestry and Food Engineering, Yibin University Yibin China; 6 Department of Biology, School of Science, King Mongkut’s Institute of Technology Ladkrabang (KMITL), Bangkok, Thailand Department of Biology, School of Science, King Mongkut’s Institute of Technology Ladkrabang (KMITL) Bangkok Thailand; 7 UC Herbarium, University of California at Berkeley, Berkeley, CA 94720-2465, USA UC Herbarium, University of California at Berkeley Berkeley United States of America; 8 School of Science, Mae Fah Luang University, Chiang Rai 57100, Thailand School of Science, Mae Fah Luang University Chiang Rai Thailand

**Keywords:** *
Agaricales
*, *
Basidiomycota
*, diversity, phylogeny, Southeast Asia, taxonomy

## Abstract

Based on unique morphological characters and molecular phylogenetic analysis, *Lasiolepiota* is described as a new genus comprising two new species, *La. oinochroa* and *La. thailandica*, collected from the forests of Laos and Thailand. *Lasiolepiota* is characterized by a glabrous patch at pileus center, small to large basidiomata covered with a thick layer of fibrous felt or woolly floccules, free and white lamellae, a fragile annulus, a similar pileus- and stipe covering a complex hyphal type of structure composed of irregular and branched hyphae, hyaline, ellipsoid, ovoid to amygdaliform basidiospores, clavate to fusiform cheilocystidia, and no pleurocystidia. Furthermore, the molecular phylogenetic analysis of combined gene sequences (ITS, LSU, *rpb*2, and *tef*1-α) confirmed that *Lasiolepiota* is a part of the *Agaricaceae* s.str., and distinctly separate from other genera.

## Introduction

The *Agaricaceae* Chevall. is diverse, species-rich, and widespread in all climate zones. Its species are saprotrophic and can be found in forests, grasslands, and dry environments. The concept of the family has changed over time; in the past it was comprised of the type of genus *Agaricus* Chevall. (plus, its satellite genera) and the lepiotoid genera (e.g., Singer, 1986); a much wider concept based on phylogenetic analyses of the LSU region, followed and included black-spored *Coprinus comatus* (O.F. Müll.) Pers., and *Montagnea* Fr., gastroid genera such as *Lycoperdon* Pers., *Calvatia* Fr., *Tulostoma* Pers., and *Podaxis* Desv., but *Cystoagaricus* Singer (now in Psathyrellaceae; Vellinga 2004), *Cystoderma* Fayod, and *Phaeolepiota* Maire ex Konrad & Maubl. were excluded (e.g., Moncalvo et al. 2002; Matheny et al. 2007; He et al. 2019). Later again, a slimmer version of the family was proposed, by definition containing brown-spored *Agaricus*, but also white-spored genera such as *Macrolepiota*, *Chlorophyllum*, *Leucoagaricus*, and *Leucocoprinus*, but not *Lepiota* itself, nor the gastroid genera mentioned above (Kalichman et al. 2020). Some genera in *Agaricaceae* were proposed based on fieldwork and phylogenetic-molecular research in Southeast Asia, including South China, viz., *Coniolepiota* Vellinga and *Eriocybe* Vellinga (Vellinga et al. 2011), and *Pseudolepiota* Z.W. Ge from southwest China (Ge and Yang 2017). Since then, the following genera were proposed: the secotioid *Asperosporus* (Karlsen-Ayala et al. (2021); Yang et al. (2024a) established *Candelolepiota* Kun L. Yang, Jia Y. Lin & Zhu L. Yang and *Macropsalliota* Kun L. Yang, Jia Y. Lin & Zhu L. Yang within the *Leucoagaricus*/ *Leucocoprinus* clade by considering differences in molecular data and their characteristics, and Radnóti et al. (2025) established *Mystagaricus* V. Papp, Radnóti & Dima based on the type species *Leucoagaricus brunneolilacinus* Babos. Furthermore, Yang et al. (2024b) transferred *Heinemannomyces* Watling into *Hymenagaricus* Heinem., in short, twenty-one genera are recognized in the *Agaricaceae* s.str.

During ongoing research on lepiotoid genera in the *Agaricaceae* in Thailand and Laos, several specimens, resembling *Lepiota fuscovinacea* F.H. Møller & J.E. Lange that do not fit in existing genera, were discovered. These were included in phylogenetic analyses but not described, as we had only one collection for each of them (Vellinga et al. 2011). Here, based on more collections from this region, we focus on one of these, describing a new genus with two new species, and providing full descriptions with discussions of their unique morphological characteristics and present phylogenetic analyses based on multiple gene regions.

## Materials and methods

### Sample collection and morphological study

In Laos, a specimen was collected in Vientiane capital and deposited in the National Herbarium of Laos (**HNL**). In Thailand, samples were collected from Chiang Rai and Chiang Mai (Northern Thailand), and Nakhon Nayok provinces (Central Thailand); and samples were deposited in the Herbarium of Mae Fah Luang University (**MFLU**), BIOTEC Bangkok Herbarium (**BBH**) and the University Herbarium at the University of California at Berkeley (**UC**).

The macromorphology was examined and observed from fresh samples. The color annotations that followed were by Kornerup and Wanscher (1978). The micromorphology was illustrated from dried material using a drawing tube attached to an Olympus CX-41 research compound microscope. The original color of all microscopic characteristics was observed in water (H_2_O) and 3–10% of potassium hydroxide (KOH), and chemical reactions were observed using Melzer’s reagent, Cotton blue, and Cresyl blue; line drawings were made of material mounted in Congo red in ammonia. The notation [x/y/z] indicates that measurements were made on x basidiospores in y individuals in z collections. The quotient (Q) of the average length and width was calculated to indicate the basidiospore shape. The technical terms used for the descriptions followed those of Vellinga (2001).

### Phylogenetic analysis

DNA was extracted from dried herbarium collections according to the instructions of the Biospin Fungus Genomic DNA Extraction Kit (Bioer Technology Co., Ltd., Hangzhou, China). The PCR amplification used ITS1 and ITS4 primers for the nrITS internal transcribed spacer regions (ITS) (White et al. 1990; Gardes and Bruns 1993), LR0R and LR5 primers for the large subunit region (LSU) (Vilgalys and Hester 1990; Rehner and Samuels 1994), fRPB2-6F and fRPB2-7R primers for polymerase II second largest subunit region (*rpb*2) (Liu et al. 1999), EF1-983F and EF1-1567R primers for translation elongation factor 1-alpha (*tef*1-α) (Rehner and Buckley 2005). The PCR amplified products were cleaned and sequenced by Shanghai Majorbio Bio-Pharm Technology Co., Ltd. (Shanghai, China). Sequences were edited and assembled using the SeqMan program (DNAStar, Madison, WI, USA), and all new sequences were deposited in GenBank. The sequences were checked against existing sequences at GenBank, and representative sequences of each genus and species of *Agaricaceae* s.str. were obtained for the analysis. One hundred forty-three specimens were used for the analysis (Table 1). The dataset of each locus was aligned using MAFFT version 7.130-win32 (Katoh et al. 2002; Katoh and Toh 2008). A Maximum likelihood (ML) analysis was performed in RAxML 7.2.6 (Stamatakis et al. 2008), with GTRGAMMA+I as the model of evolution, and branch support was estimated to be over 1,000 bootstrap partitions (BP) with the rapid bootstrap option. A Bayesian inference (BI) analysis was performed with MrBayes 3.2.7a (Ronquist and Huelsenbeck 2003) in the CIPRES webserver. The best substitution model of the individual dataset was defined by using MrModelTest v.2.3 (Nylander 2004), and the best model for all datasets were GTR+I+G. For the BI analysis, four Metropolis-coupled Markov chain Monte Carlo (MCMC) runs, each with four heated chains and two cold chains, and the run was conducted for 20 million generations and sampled every 1000 generations, with the first 25% discarded as burn-in. The phylogram results of all analyses were exported and edited in TreeView1.0.0.0 (Page 1996). The phylogenetic tree was edited for publication using Adobe Illustrator CS6 (Adobe Systems Incorporated, USA).

**Table 1.** Sequences used in this study and their GenBank accession numbers. New additions in bold.

**Species**	**Voucher no**.	**Country**	**GenBank accession no**.			
			**ITS**	**LSU**	***rpb2***	***tef*1-α**
*Agaricus campestris*	LAPAG370	China	KM657927	KR006607	KT951556	KR006636
*Agaricus diminutivus*	ecv2360	USA	AF482831	AF482877	–	–
*Agaricus friesianus*	ZRL2012601	China	KX657026	KX656970	KX685048	KX684963
*Agaricus gemloides*	ZRL2012003	China	KX657022	KX656958	KX685063	KX684954
*Agaricus gratolens*	ZRL3093	Thailand	JF691548	KT951488	KT951560	–
*Agaricus inapertus*	ecv2339	USA	AF482834	AF482878	–	HM488878
*Agaricus parvibicolor*	ZRL2012029	China	KX657018	KX656960	KX685060	KX684956
*Agaricus purpurellus*	ZRL2012588	China	KX657025	KX656968	KX685050	KX684961
*Agaricus* sp.	ZRL2012199	China	KT951367	KT951470	KT951537	KT951595
*Asperosporus subterraneus*	FLAS-F-68001	USA	MW775328	MW773843	MW816922	MW816921
*Barcheria willisiana*	H7518	Australia	JF495037	JF495020	–	–
*Barcheria willisiana*	H7564	Australia	JF495038	JF495019	–	–
*Barcheria willisiana*	MEL2177563	Australia	JF495036	AY372216	–	–
*Candelolepiota sinica*	HMAS60647	China	DQ182505	DQ457653	–	–
*Candelolepiota sinica*	HTBM2008	China	PQ321874	PQ319800	PQ329229	PQ329237
*Candelolepiota sinica*	HTBM1241	China	PQ321882	PQ319808	PQ329236	PQ329247
*Chlorolepiota brunneotincta*	CN5221	India	JQ928940	–	–	–
*Chlorophyllum africanum*	PREM 62140	South Africa	MG741961	MG742041	MG742070	MG742098
*Chlorophyllum africanum*	PREM 62141	South Africa	MG741963	MG742042	MG742071	MG742099
*Chlorophyllum agaricoides*	HKAS 101312	Russia	MG742003	MG742020	MG742050	MG742078
*Chlorophyllum agaricoides*	HMAS 71678	China	MG742004	MG742021	MG742051	MG742079
*Chlorophyllum brunneum*	HKAS 101315	Italy	MG742013	MG742022	MG742052	MG742080
*Chlorophyllum demangei*	Z. W. Ge 3112	China	MG741965	MG742027	MG742056	MG742084
*Chlorophyllum globosum*	PREM 62147	South Africa	MG742002	MG742024	MG742053	MG742081
*Chlorophyllum hortense*	HKAS 101317	China	MG741967	MG742026	MG742054	MG742082
*Chlorophyllum molybdites*	HKAS 45051	China	MG741985	MG742030	MG742059	MG742087
*Chlorophyllum molybdites*	Z. W. Ge 3377	USA	MG741992	MG742033	MG742062	MG742090
*Chlorophyllum neomastoideum*	HKAS 83208	China	MG741976	MG742035	MG742064	MG742092
*Chlorophyllum sphaerosporum*	HMAS 66153	China	MG742011	MG742043	MG742072	MG742100
*Clarkeinda trachodes*	ecv3550	Thailand	HM488751	–	HM488802	–
*Clarkeinda trachodes*	ecv3838	Thailand	HM488750	HM488771	–	–
*Clarkeinda trachodes*	HKAS122726	China	ON794453	–	–	–
*Clarkeinda trachodes*	KUBOT-KRMK-2020-26	India	MW425600	MW425592	–	–
*Coniolepiota spongodes*	png012	Thailand	HM488756	HM488774	HM488796	HM488883
*Coniolepiota spongodes*	ecv3898	Thailand	HM488755	–	HM488798	HM488882
*Coniolepiota spongodes*	ecv3613	Thailand	HM488757	–	HM488797	HM488881
*Coniolepiota spongodes*	HKAS:77574	Bangladesh	KC625531	KC625530	–	–
*Eriocybe chionea*	ecv3616	Thailand	HM488753	HM488772	HM488801	–
*Eriocybe chionea*	ecv3560	Thailand	HM488752	HM488773	HM488800	–
*Hymenagaricus splendidissimus*	HTBM1385	China	PP736734	PP732964	PP746792	PP746820
*Hymenagaricus splendidissimus*	ZRL185	Thailand	KT951346	KT951527	–	KT951657
*Hymenagaricus splendidissimus*	ecv3586	Thailand	HM488760	HM488769	HM488793	–
*Hymenagaricus splendidissimus*	GDGM:46633	China	MF621038	MF621039	–	–
*Hymenagaricus splendidissimus*	Q. Zhao 2591	China	KY039571	KY039576	–	–
*Hymenagaricus splendidissimus*	Z.W. Ge 2540	China	KY039570	KY039575	–	–
*Hymenagaricus ardosiicolor*	Z4	Tanzania	KM360160	–	–	–
*Hymenagaricus brunneodiscus*	LAH37956	Pakistan	OR510862	OR510864	–	–
Hymenagaricus cf. kivuensis	BR6089	Burundi	KM982454	–	–	–
*Hymenagaricus pakistanicus*	FAK195	Pakistan	OP082404	–	–	–
*Hymenagaricus saisamornae*	SDBR-CMUNK0369	Thailand	MW345912	MW345917	–	–
*Hymenagaricus saisamornae*	SDBR-CMUNK0567	Thailand	MW349602	MW349604	–	–
*Hymenagaricus* sp.	ZRL3103	Thailand	KM982450	KM982452	–	–
*Hymenagaricus* sp.	LD2012186	Thailand	KM982451	KM982453	–	–
***Lasiolepiota oinochroa***	**STO-2019-398**	**Thailand**	**OP297176**	**OP295046**	**OP321133**	**OP321131**
***Lasiolepiota oinochroa***	**HNL503339**	**Laos**	**OP297177**	**OP295047**	**OP321132**	**OP321130**
***Lasiolepiota thailandica***	**BBH49568**	**Thailand**	**PZ324390**	**PZ324391**	–	–
***Lasiolepiota thailandica***	**ecv3556**	**Thailand**	**HM488759**	**HM488776**	–	–
Lepiota aff. fuscovinacea	MFLU 09-00129	Thailand	HM488758	HM488775	–	–
*Leucoagaricus albosquamosus*	HMAS287858	China	PP816118	PQ618995	PQ634990	–
*Leucoagaricus badius*	SH148	Pakistan	KU647736	KU900518	KU900506	KU900510
*Leucoagaricus centrobrunneolus*	HMAS287820	China	PP816117	PP816156	PQ305432	PQ356767
*Leucoagaricus croceus*	MFLU 19-1331	Thailand	OL413492	MT515802	MT518174	–
*Leucoagaricus lahorensiformis*	SHL2	Pakistan	KU647729	KU900517	KU900509	KU900511
*Leucoagaricus rubrotinctus*	KUN:HKAS 54317	China	JN944082	JN940294	JN993685	–
*Leucoagaricus rubrotinctus*	KUN:HKAS 54240	China	JN944081	JN940295	JN993684	–
*Leucoagaricus sultanii*	SH115	Pakistan	KU647733	KU900519	KY965810	KU900513
*Leucoagaricus pakistaniensis*	SJF13	Pakistan	KU647727	KU900515	KY965808	KU900512
*Leucoagaricus atroviridis*	HMAS287845	China	PP816130	PQ618980	PQ634979	–
*Leucocoprinus attinorum*	QVM2	Brazil	MN473885	MN473137	–	MN482668
*Leucoagaricus barssii*	ECV 3126	USA	DQ911600	DQ911601	DQ911602	GU187722
*Leucocoprinus birnbaumi*	HUH#SH9	Pakistan	KJ717764	AF041541	–	–
*Leucocoprinus cepistipes*	xml2014128	China	LT716023	KY418838	KY418990	KY419045
*Leucocoprinus cretaceus*	HTBM0214	China	PQ321877	PQ319803	PQ329232	PQ329240
*Leucocoprinus cretaceus*	Laessoe6171	Malaysia	AF482861	AF482892	–	–
*Leucocoprinus fragilissimus*	ZRL20151466	China	LT716029	KY418844	KY418994	KY419049
*Leucocoprinus licmophorus*	HTBM0689	China	PQ321879	PQ319805	PQ329233	PQ329244
*Leucocoprinus leucothites*	KUN:HMAS 86785	Kingdom	JN944084	JN940292	JN993694	–
*Leucocoprinus nympharum*	KUN:HKAS 61621	China	JN944085	JN940289	JN993690	–
*Leucocoprinus beijingensis*	HMAS287803	China	PP816074	PP816169	PQ305416	PQ305437
*Leucocoprinus tangerinus*	HTBM0679	China	PQ321878	PQ319804	–	PQ329243
*Macrolepiota dolichaula*	AFTOL-ID 481	China	DQ221111	–	DQ385886	DQ435785
*Macrolepiota dolichaula*	KUN:HKAS 50547	China	–	JN940269	JN993700	–
*Macrolepiota mastoidea*	H.A. Huijser 6266 (herb. Huijser)	Belgium	–	–	HM488863	HM488894
*Macrolepiota mastoidea*	KUN:HKAS 11084	China	–	JN940271	JN993701	–
*Macrolepiota mastoidea*	KUN:HKAS 11207	China	–	JN940272	JN993698	–
*Macrolepiota procera*	KUN:HKAS 8108	China	–	JN940277	JN993697	–
*Macrolepiota procera*	KUN:HKAS 5722	China	–	JN940275	JN993696	–
*Macrolepiota sabulosa*	UFRN-Fungos 2693	Brazil	KY927715	KY907185	KY964598	–
*Macrolepiota* sp.	Thomas Laessoe tl9329 (C)	–	–	–	HM488866	HM488891
*Macrolepiota* sp.	UFRN-Fungos 2692	Brazil	KY927723	KY907193	KY964603	–
*Macrolepiota* sp.	UFRN-Fungos 2691	Brazil	KY927722	KY907192	KY964602	–
*Macrolepiota* sp.	UFRN-Fungos 2690		KY927721	KY907191	KY964601	–
*Macropsalliota americana*	CCBAS802	Czech Republic	LN714565	–	LN714673	–
*Macropsalliota americana*	NYBG:0005		KY350857	KY350858	–	–
*Macropsalliota americana*	ecv2454		AY176407	AF482891	–	–
*Macropsalliota meleagris*	ecv1990		AY176419	AF482890	–	–
*Macropsalliota meleagris*	AJ 542		MN483027	–	–	–
*Macropsalliota meleagris*	FLAS-F-60750		MH016840	–	–	–
*Macropsalliota mucrocystis*	DB-FG-167-19		MZ047586	MZ047585	–	–
*Macropsalliota mucrocystis*	CA 16		MN483026	–	–	–
*Macropsalliota subtropica*	ZRL20233628		PP816132	PQ618986	PQ634974	–
*Macropsalliota subtropica*	HKAS 124380		OP339899	OP339901	–	–
*Macropsalliota tropica*	CUH AM699		MT669365	MT669370	–	–
*Macropsalliota tropica*	CUH AM707		MT669367	MT669371	–	–
*Macropsalliota tropica*	HAW-F-00249		MK412616	–	–	–
*Micropsalliota bifida*	HFJAU2998	China	OM650272	OM650252	OM669858	–
*Micropsalliota cortinata*	HFJAU0713	China	MN508426	OM650253	OM669874	–
*Micropsalliota furfuracea*	HFJAU3123	China	OM650276	OM650254	OM669873	–
*Micropsalliota globocystis*	ZRL2013465	China	LT716024	KY418839	KY418991	KY419046
*Micropsalliota globocystis*	HFJAU2709	China	OM650278	OM650262	OM669856	–
*Micropsalliota globocystis*	HFJAU1518	China	OM650277	OM650255	OM669852	–
*Micropsalliota megaspora*	HFJAU1255	China	OM650282	OM650258	OM669876	–
*Micropsalliota pseudodelicatula*	HFJAU2228	China	OM650288	OM650264	OM669863	–
*Micropsalliota pusillissima*	SFSU zrl 3047	Thailand	HM436645	HM436594	–	–
*Micropsalliota xanthorubescens*	SFSU zrl 3083	Thailand	HM436638	HM436598	–	–
*Micropsalliota* sp.	ecv3638	Thailand	HM488762	HM488768	HM488794	–
*Mystagaricus brunneolilacinus*	DB-2023–06-09–1	Austria	PV659895	PV659888	PV657075	PV661643
*Mystagaricus brunneolilacinus*	VPapp-23032601	Hungary	PV659894	PV659887	PV657074	PV661642
*Mystagaricus brunneolilacinus*	JDRG-31071001	Spain	OK582196	–	–	–
*Podaxis pistillaris*	Pdxc	India	MN134342	MN134344	–	–
*Podaxis rugospora*	PREM 27280	South Africa	KT844863	KT844827	–	–
*Podaxis rugospora*	PREM 14507	Southern Africa	KT844860	KT844821	–	–
*Podaxis* sp.	PREM 60320	Southern Africa	KT844879	KT844848	–	–
*Podaxis* sp.	PREM 34405	Southern Africa	KT844877	KT844831	–	–
*Pseudolepiota zangmui*	Z. W. Ge 2175	China: Yunnan	KY768928	MG742049	KY768929	MG742106
*Pseudolepiota zangmui*	Z. W. Ge 2107	China: Yunnan	KY768927	–	KY768930	–
*Pseudolepiota zangmui*	MFLU 10-0515	Thailand	KX904355	–	–	–
*Termiticola* sp.	TL6106	Malaysia	AY176352	AY176353	–	–
*Termiticola* sp.	HKAS122480	China	ON794316	–	–	–
*Termiticola* sp.	rlc-381	China	MW374173	–	–	–
*Termiticola* sp.	WML-08	China	OK584457	–	–	–
*Xanthagaricus caeruleus*	GDGM:50651	China	MF039088	MF039086	–	–
*Xanthagaricus epipastus*	SFSU zrl 3045	Thailand	HM436649	HM436609	–	–
*Xanthagaricus erinaceus*	HTBM0936	China	PP736704	PP732934	PP746788	–
*Xanthagaricus flavosquamosus*	GDGM:50918	China	MF351629	MF351631	–	–
*Xanthagaricus heinemannii*	HKAS133458	China	PP736714	PP732944	PP746799	PP746829
*Xanthagaricus necopinatus*	Iqbal821	Bangladesh	MF351626	MF351630	–	–
*Xanthagaricus purpureosquamulosus*	MFLU 19-2354	Thailand	MN099353	MN097917	–	–
*Xanthagaricus purpureosquamulosus*	HTBM1932	China	PP736667	PP732897	PP746801	PP746832
*Xanthagaricus retisporus*	HTBM1870	China	PP736662	PP732892	PP746797	PP746826
*Xanthagaricus siamensis*	MFLU 19-0574	Thailand	MN176992	MN176982	–	–
*Xanthagaricus siamensis*	HNL503338	Laos	ON063325	ON063326	ON083789	–
*Xanthagaricus taiwanensis*	AFTOL-ID 1383	China	DQ490633	DQ457680	–	–
*Xanthagaricus thailandensis*	DSBR-MUJK0115	Thailand	MG256664	MG256666	–	–
*Echinoderma asperum* (outgroup)	KUN-HKAS 105694	China	MN810153	MN810106	MN820966	MN820908
*Lepiota maculans* (outgroup)	JMB 05-08-09-18	USA	HM222939	HQ832458	HQ832436	–

## Results

### Phylogenetic analysis

The final combined dataset of ITS-LSU-*rpb*2-*trf*1-α genes was 2919 characters (including gaps), which comprised 138 collections and 702 characters of ITS, 124 collections and 908 characters of LSU, 84 collections and 738 characters of *rpb*2, 53 collections and 571 characters of *tef*1-α. *Echinoderma asperum* (Pers.) Bon and *Lepiota maculans* Peck (Verrucosporaceae) were used as the outgroup. The best RaxML tree with a final likelihood value of −52296.311392 is presented. The matrix had 1763 distinct alignment patterns, with 35.01% undetermined characters or gaps. Estimated base frequencies were as follows: A = 0.236207, C = 0.222605, G = 0.243147, T = 0.298041; substitution rates AC = 1.340209, AG = 4.947081, AT = 1.391667, CG = 0.808856, CT = 5.882094, GT = 1.000000; gamma distribution shape parameter α = 0.282742. The phylogenetic tree derived from the Bayesian analysis was similar to that derived from the ML analysis.

**Figure 1.**
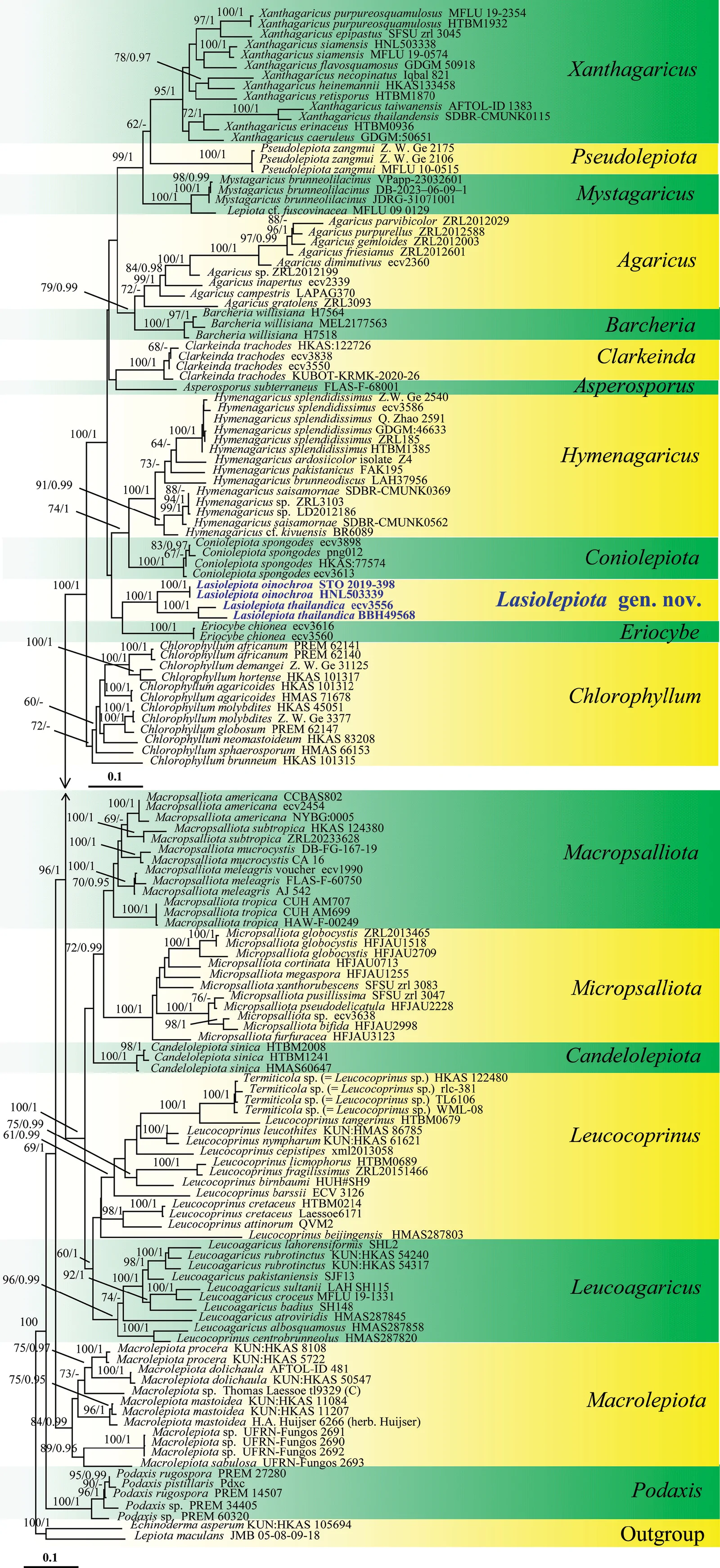
Maximum Likelihood tree of *Agaricaceae* s.str. based on Maximum Likelihood analysis of ITS-LSU-*rpb2*-*tef*1-α sequence data. Bootstrap values for Maximum Likelihood equal to or greater than ≥ 60% and Bayesian posterior probabilities ≥ 0.95 are provided above the branches (ML/BI). *Echinoderma asperum* and *Lepiota maculans* were used as outgroups. New sequences are in blue.

The results mostly supported genera being separated into distinct subclades. However, there is an unresolved clade consisting of two genera, viz., *Leucocoprinus* and *Termiticola* (Fig. 1). In our focus clade, *Lasiolepiota* has high bootstrap support (BS) (100%) and posterior probability (PP) (1.00), consisting of two new species, and is sister to *Eriocybe*; these two genera with *Hymenagaricus* and *Coniolepiota*, form a separate clade but without significant support. For this analysis, representative taxa of each genus in *Agaricaceae* s.str. were selected, resulting in the representation of twenty-one mainly agaricoid genera. The combined dataset of ITS-LSU-*rpb*2-*tef*1-α was analyzed.

### Taxonomy

#### Lasiolepiota

Taxon classificationFungi

Sysouph., Yuan S. Liu & Vellinga gen. nov.

EECE23B7-DB3C-53AD-9F66-12BD9B6C7D18

844795

##### Diagnosis.

***Basidiomata*** lepiotoid, fragile. ***Pileus*** subumbonate to plano-concave, with straight margin; with an irregular glabrous, persistent, grayish ruby to dark ruby patch at center, covered with a thick layer of fibrous felt or woolly floccules on the whole surface, violet brown to dark ruby; with concolorous felt as on surface and exceeding lamellae. ***Lamellae*** free, white, crowded, broadly ventricose. ***Stipe*** cylindrical to subcylindrical, covered with a violet brown to dark ruby layer of fibrous felt, hollow. ***Annulus*** membranous. ***Basidiospores*** ellipsoid ovoid to amydaliform, hyaline. ***Basidia*** short and clavate, hyaline, 4-spored. ***Cheilocystidia*** clavate, fusiform or utriform, hyaline, branched. ***Pleurocystidia*** absent. Pileus- and stipe covering an irregular cutis composed of cylindrical hyphae and elements. ***Clamp connections*** absent.

##### Typus generi.

*Lasiolepiota oinochroa* Sysouph., Yuan S. Liu & Vellinga.

##### Etymology.

from the combination of Greek “λασιοτριχος” (lasiotrichos, meaning “shaggy”) and the genus name *Lepiota*, which looks like the genus *Lepiota* but the basidiomata are shaggy-haired.

#### Lasiolepiota oinochroa

Taxon classificationFungi

Sysouph. & Yuan S. Liu sp. nov.

C54C0B51-24FC-58A0-A187-EB76099F2DC1

844796

Figs 2, 3

##### Etymology.

The specific epithet “oinochroa” is from the Greek “οινοχρωος” (oinochroos, meaning “wine-coloured), because basidiomata are fully covered with wine-coloured shaggy hairs.

**Figure 2.**
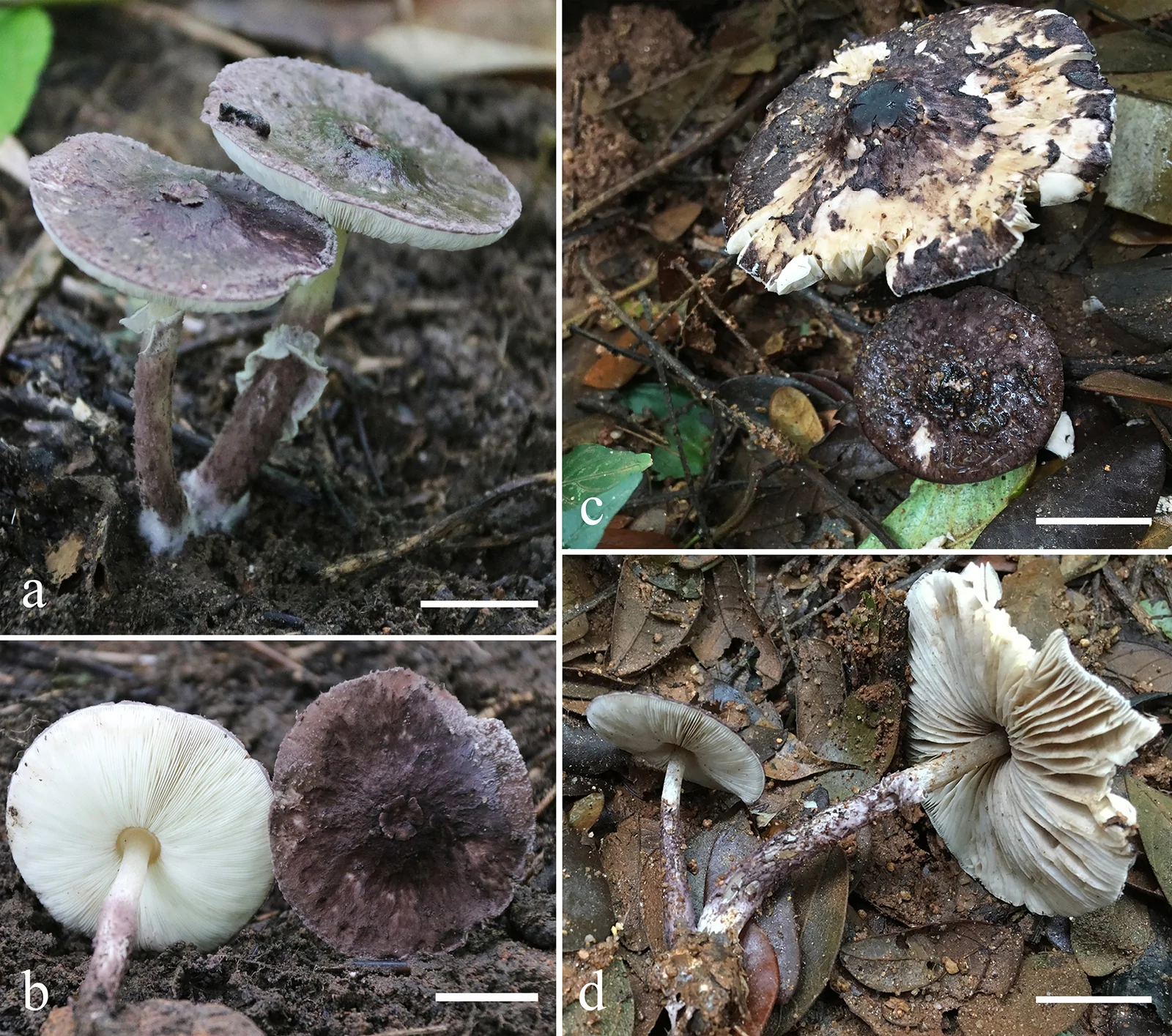
Fresh basidiomata of *Lasiolepiota oinochroa* in situ. **a, b**. HNL503339 (holotype); **c, d**. STO-2029-308 (paratype). Scale bars: 20 mm (**a–d**).

**Figure 3.**
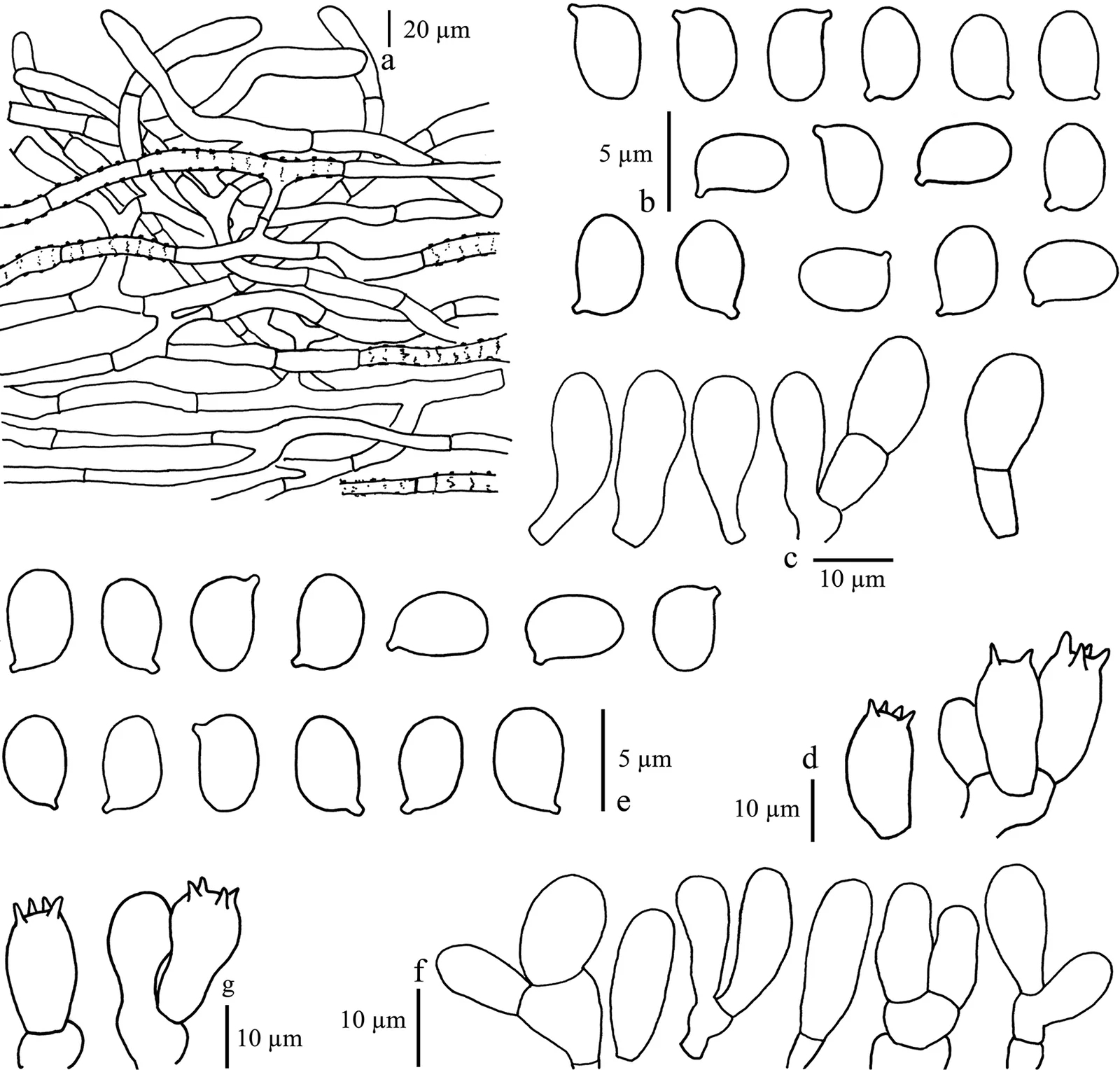
Microcharacters of *Lasiolepiota oinochroa*. **a**. Pileus covering made up of elements cylindrical elements with smooth and encrusted walls; **b, e**. Basidiospores; **c, f**. Cheilocystidia; **d, g**. Basidia; **a–d**. HNL503339 (holotype); **e–g**. STO-2019-398.

##### Holotype.

Laos • Vientiane Capital, Xaythany district, Houay Nhang Preserves Forest, 29 July 2016, collected by Phongeun Sysouphanthong (HNL503339, holotype).

##### Description.

***Basidiomata*** small to medium-sized. ***Pileus*** 35–65 mm diam., subumbonate to umbonate, expanding to applanate or slightly plano-concave, with straight margin, with an irregular patch at center or umbo, glabrous, persistent, grayish ruby (12E5–7) to dark ruby (12F4–8), around patch covered with a thick layer of fibrous felt or woolly, very crowded, violet brown (11F–8) to dark ruby (12F5–8), darker when wet, floccules towards margin, on white to yellowish white background (4–5A2); margin with concolorous felt as on surface and exceeding lamellae, with irregular velar remnants or appendiculate, remnants fragile when mature. ***Lamellae*** free, remote from stipe, white to yellowish white with age, very crowded, broadly ventricose, up to 6 mm wide, more than 5 series of lamellulae, with concolorous, eroded lamella edge. ***Stipe*** 43–80 × 3.5–6.0 mm, cylindrical or slightly wider at base and tapering to apex, with white fibrillose background, white fibrillose from apex to annulus, covered with concolorous fibrous felt as on pileus below annulus down towards base, with white fibrous rhizomorphs. ***Annulus*** membranous, hanging down, white on upper side and covered with concolorous fibrous felt as on stipe surface at underside, sometimes fragile or disappearing when mature. ***Context*** white and up to 3 mm thick in pileus, in stipe white, hollow, not discoloring, dull white with age. Taste and odor not observed. Spore print white.

***Basidiospores*** [75/2/2] l × w = 4.5–5.0 × 3.0–4.0 μm, al × aw = 4.84 × 3.44 μm, Q = 1.3–1.5, Qav = 1.41, ellipsoid ovoid to amygdaliform in side view, in frontal view ellipsoid, smooth, hyaline, slightly thick-walled, dextrinoid, congophilous, cyanophilous in cotton blue, not metachromatic in Cresyl blue. ***Basidia*** (n = 20) 12–15 × 5.0–6.0 μm, short clavate, hyaline, 4-spored, often 2-spored. ***Lamella trama*** regular to slightly irregular. ***Pleurocystidia*** absent. ***Cheilocystidia*** (n = 20) 10–28 × 6.0–10.0 μm, short clavate to clavate, sometimes fusiform or utriform, hyaline, sometimes branched. ***Pileus covering*** complex, made up of interwoven branched and cylindrical hyphae; hyphae (n = 15) 3.0–10 μm wide, terminal elements (n = 20) 55–85 × 3.5–7.5 μm, cylindrical, sometimes with incrusting, often with violet parietal and intracellular pigments. ***Stipe covering*** similar to pileus covering. ***Clamp connections*** absent.

##### Habitat and distribution.

On sandy loam of deciduous forests with mixed tree species. Known from central Laos, and northern Thailand.

##### Additional material examined.

Thailand • Chiang Rai province, Muang district, Mae Fah Luang University Campus, 06 August 2019, collected by Yuan S. Liu (STO-2019-398, paratype).

##### Note.

Two collections of *Lasiolepiota oinochroa* were found, one in Laos and one in Thailand. When we compared their morphology and molecular analysis, they were identical (Figs 1, 2, 3). *Lepiota fuscovinacea* from temperate Europe is closely similar to *Lasiolepiota oinochroa* by its lilac-grayish to purple pileus and brown to dark-purple fibrillose squamules, but *L. fuscovinacea* has a trichodermal pileus covering and lacks a glabrous patch at the center of the pileus; furthermore, *L. fuscovinacea* belongs to *Lepiota* in *Verrucosporaceae* (Candusso and Lanzoni 1990; Vellinga 2001; Kalichman et al. 2020). *Coniolepiota spongodes* (Berk. & Broome) Vellinga is very similar to *La. oinochroa* by morphology, viz., pileus and stipe are covered with lilac to purple-gray powdery velar warts, white lamellae, thin membranous to cortina-liked annulus; but *Co. spongodes* is different as it lacks a glabrous patch at the pileus, center, longer basidiospores (4.4–6.4 × 2.7–3.7 μm, Q = 1.4–1.8), the pileus covering is powdery and the velar warts are made up of cylindrical to irregular, branched hyphae (t-shaped or with more side branches, three-dimensional structure) (Vellinga et al. 2011). *Mystagaricus brunneolilacinus* (Babos) V. Papp, Radnóti & Dima (= *Leucoagaricus brunneolilacinus* Babos) differs from *La. oinochroa* by the absence of a glabrous patch at the center of the pileus, covering with brownish lilac to brownish purple-lilac, purplish brown to vinaceous woolly to fibrillose on pileus and stipe (Babos 1980; Radnóti et al. 2025).

#### Lasiolepiota thailandica

Taxon classificationFungi

Wisitrassameewong, Sysouph. & Vellinga sp. nov.

05B49F51-A676-5523-AAD1-64FBBFC4A646

863875

Figs 4, 5

##### Etymology.

“thailandica” from the country of the holotype “Thailand”.

**Figure 4.**
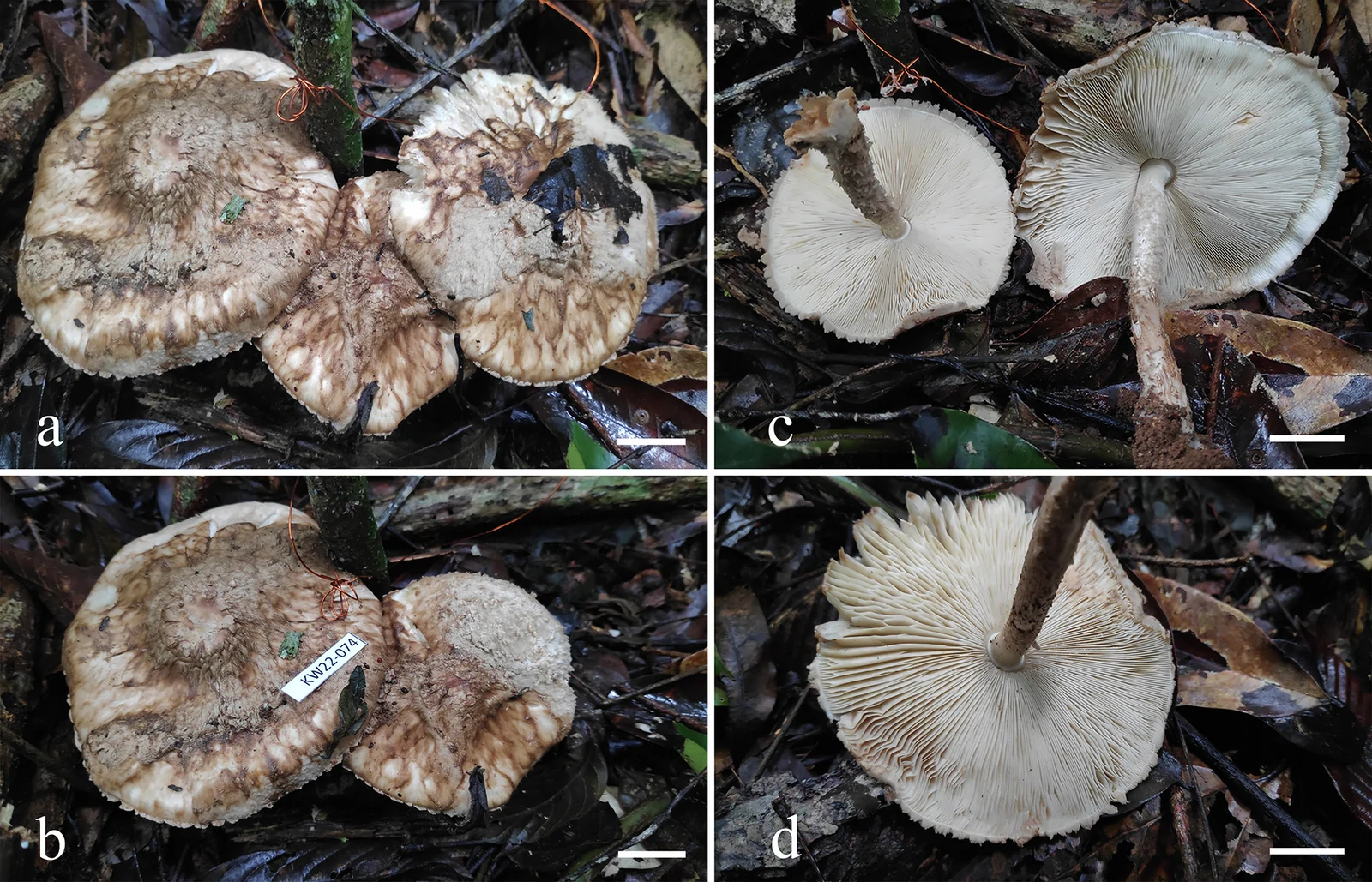
Fresh basidiomata of *Lasiolepiota thailandica* in situ. **a–d**. BBH49568 (holotype). Scale bars: 20 mm (**a–d**).

**Figure 5.**
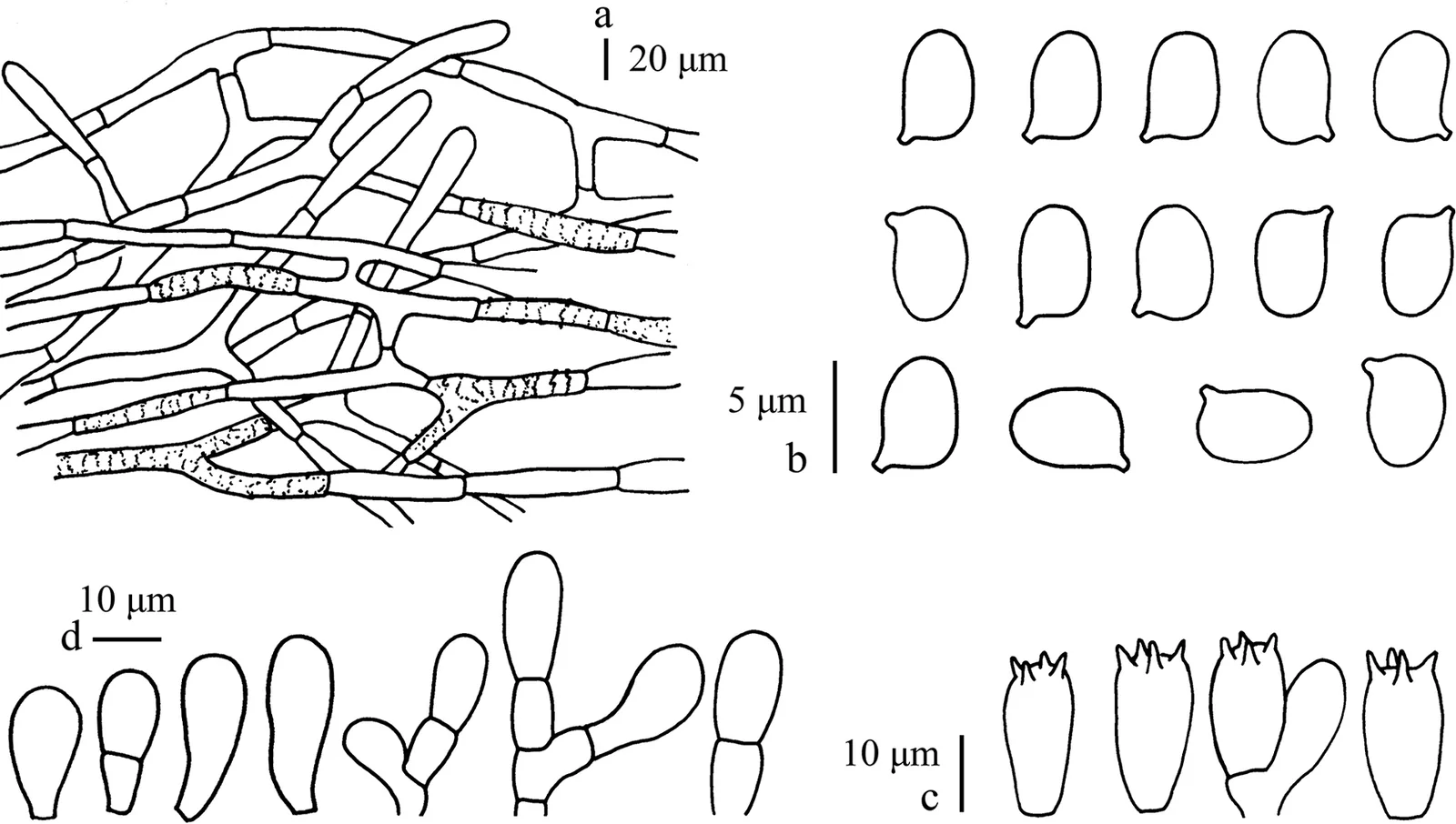
Microcharacters of *Lasiolepiota thailandica* (BBH49568, holotype). **a**. Pileus covering made up of elements cylindrical elements with smooth and encrusted walls; **b**. Basidiospores; **c**. Basidia and basidioles; **d**. Cheilocystidia.

##### Holotype.

Thailand • Nakhon Nayok Province, Khao Yai National Park, 03 August 2022, collected by Komsit Wisitrassameewong (BBH49568, holotype).

##### Description.

***Basidiomata*** medium to large-sized. ***Pileus*** up to 115 mm diam., convex, expanding to umbonate to plano-concave, with straight to deflexed margin; with an irregular patch at center or umbo, glabrous, persistent, reddish white (9A2–3) to reddish gray to dull red (9B2–3), around patch covered with a thick layer of fibrous felt or woolly floccules towards the margin, crowded, reddish gray to dull red (8B2–3) to brownish gray (8C2–3), darker when wet, reddish brown (8D4–6), on white background; margin felted, with velar remnants or appendiculate, reddish gray (8B2). ***Lamellae*** free, remote from the stipe, white, very crowded, broadly ventricose, up to 5 mm wide, with up to 3 series of lamellulae, with concolorous eroded lamella edge, turning reddish white to pale red (8A2–3) when touched. ***Stipe*** 85–110 × 6–10 mm, cylindrical or slightly wider towards base, with white background at the top to the annulus zone, below the annulus covered with a layer of fibrous felt or woolly floccules as on pileus, reddish gray to dull red (8B2–3) from apex to annular zone, brownish gray (8C2–3) to reddish brown (8D4–6) from annular zone down towards base, with white fibrous rhizomorphs. ***Annulus*** hanging down, white on the upper side and covered with concolorous fibrous felt as on the stipe surface at underside, sometimes fragile or disappearing when mature. ***Context*** white and thick in pileus, up to 10 mm thick, turning reddish white to pale red (8A2–3) when cut or darker when dry, in stipe white, hollow, turning color as in pileus, dull white with age. Taste and odor not observed. Spore print white.

***Basidiospores*** [50/1/1] l × w = 4.5–5.0 × 3.2–3.8 μm, al × aw = 4.904 × 3.41 μm, Q = 1.26–1.66, Qav = 1.44, ellipsoid ovoid to amygdaliform in side view, in frontal view ellipsoid, smooth, hyaline, slightly thick-walled, dextrinoid, congophilous, cyanophilous in cotton blue, not metachromatic in Cresyl blue. ***Basidia*** (n = 20) 15–20 × 7.5–10 μm, short clavate, hyaline, 4-spored. ***Lamella trama*** regular to slightly irregular. ***Pleurocystidia*** absent. ***Cheilocystidia*** (n = 20) 15–25 × 7–10 μm, short clavate to clavate, sometimes fusiform or utriform, rarely oblong, hyaline, sometimes branched. ***Pileus covering*** complex, made up of irregular and branched hyphae, cylindrical and interwoven; hyphae (n = 15) 8–20 μm wide; terminal elements (n = 20) 80–125 × 10–18 μm, cylindrical sometimes with incrusting, often with brown parietal and intracellular pigment. ***Stipe covering*** similar to pileus covering. ***Clamp connections*** absent.

##### Additional material examined.

Thailand • Chiang Mai province, Mae Taeng district, Pha Deng village, 25 June 2007, collected by Else C. Vellinga (ecv3556, UC)).

##### Habitat and distribution.

On humus-rich soil of deciduous forest dominated by species of Fagaceae. One collection was found in Khao Yai National Park, Nakhon Nayok Province of central Thailand, and one collection was found in Chiang Mai Province, northern Thailand.

##### Note.

*Lasiolepiota thailandica* has a distinct glabrous patch on the pileus and a broad pileus up to 115 mm. *Lasiolepiota thailandica* shares its morphology with *La. oinochroa*, but has much larger basidiomata, lighter colored patch (reddish white to reddish gray), a lighter colored layer of fibrous felt or woolly floccules (reddish gray to brownish gray) on pileus and stipe, and turning reddish white to pale red while not discoloring in *La. Oinochroa* when cut. However, their microcharacters are very similar (Figs 2, 3, 4, 5). Moreover, they clustered in the phylogenetic analysis with high BS (100%) (Fig. 1). *Mystagaricus brunneolilacinus* differs from *La. thailandica* by its smaller basidiomata, absence of a glabrous patch at the center of the pileus, and purplish brown to vinaceous pileus- and stipe covering (Radnóti et al. 2025).

## Discussion

### Phylogenetic placement

In *Agaricaceae* s.l., the combined multiple gene analysis (ITS-LSU-*rpb*2-tef1-α) has narrowed the clades of *Agaricaceae* s.str. (Kalichman et al. 2020; Karlsen-Ayala et al. 2021; Yang et al. 2024a, 2024b; Radnóti et al. 2025). For this study, sequences of representative genera of *Agaricaceae* s.str. were selected for phylogenetic analysis, and the results of our multigene analyses are consistent with the studies mentioned above. *Lasiolepiota* is the new branch of the *Agaricaceae* consisting of two new species, it has high bootstrap support (100%), and it is related to several genera occurring the same tropical region, viz., *Eriocybe*, *Coniolepiota*, and *Hymenagaricus* (Fig. 1). However, this clade received low BS based on this combined-gene analysis. *Eriocybe* is mostly related to *Lasiolepiota* with low BS, as well as other genera in this clade, and this position might change if we include the number of representatives of each genus and other species of other families. For example, Yang et al. (2024b) analyzed *Agaricaceae* sensu lato, including other families, based on combined genes of ITS-LSU-*rpb2*-*tef*1-α; they found that *Coniolepiota* and *Clarkeinda* are in the same clade with low BS, but *Eriocybe* is in another clade related to *Pseudolepiota* and *Xanthagaricus*. We suppose that more analysis with more genes might support a better result to resolve the position of genera in *Agaricaceae* s.str.

### Morphological comparison

The discovery of *Lasiolepiota* was caused by our survey of lepiotoid mushrooms in Laos and Thailand; several specimens resemble *Lepiota fuscovinacea* in the wine-colored pileus covering but differ in pileus covering structure and molecular data, and these were noted as L. aff. fuscovinacea (ecv3556) and L. cf. fuscovinacea (MFLU 09-00129) in Vellinga et al. (2011). Thanks to our surveys in under-sampled areas of Thailand and Laos, we now know that there are two species in this genus. However, the second collection (MFLU 09-00129) is still awaiting a formal description and genus placement. *Lasiolepiota* is characterized by the presence of a glabrous patch at the pileus center, basidiomata covered with a thick layer of fibrous felt or woolly floccules composed of a complex hyphal type structure made up of interwoven, irregularly branched and cylindrical hyphae, hyaline ellipsoid ovoid to amygdaliform basidiospores, clavate to fusiform cheilocystidia, and no pleurocystidia. The white-spored genus, *Eriocybe*, is very close to *Lasiolepiota* in the phylogenetic analysis (Fig. 1), but *Eriocybe* has very distinct small white basidiomata covered with cotton wool-like broad fluffy warts. *Coniolepiota spongodes* is very similar to *Lasiolepiota* by the pileus and stipe covering consisting of lilac to purple-gray powdery velar warts, white lamellae, thin, membranous to cortina-like annulus; but *Coniolepiota spongodes* lacks a glabrous patch at the center of the pileus, and the powdery velar warts are made up of short cylindrical to irregularly branched hyphae (t-shaped or with more side branches) (Vellinga et al. 2011). *Mystagaricus* differs from *Lasiolepiota* by the absence of a glabrous patch at the center of the pileus, purplish brown to vinaceous woolly to felty pileus- and stipe covering, sometimes with fibrillose scales on the pileus, whitish to cream pileus surface and turning tobacco brown when dry, but the structure of the pileus covering is not maintained in *Mystagaricus* (Radnóti et al. 2025). *Pseudolepiota* has various brown colors in the type specimens (Ge and Yang 2017), but violet-brown to dark ruby in Thai specimens (Sysouphanthong et al. 2021); *Ps. zangmui* differs from *Lasiolepiota* by its squamulose pileus and hymenodermal pileus covering structure. And lastly, *Clarkeinda* Kuntze has very large robust basidiomata, a persistent patch at the center of the pileus, but differs in greenish lamellae and yellowish spore print (Hosen and Ge 2012; Sysouphanthong et al. 2021). Our new genus has unique characteristics, and molecular evidence supports its difference from other described genera in *Agaricaceae* s.str.

## Supplementary Material

XML Treatment for Lasiolepiota

XML Treatment for Lasiolepiota oinochroa

XML Treatment for Lasiolepiota thailandica

## Additional information

### Artificial Intelligence (AI) use

The authors accept full responsibility for the content of the manuscript, including the disclosure of any use of AI.

No AI tools were used in the preparation of this manuscript.

### Author ORCIDs

P. Sysouphanthong https://orcid.org/0000-0002-1275-2459

K. Wisitrassameewong https://orcid.org/0000-0003-1195-0338

Y.S. Liu https://orcid.org/0000-0002-7902-8238

N. Tangthirasunun https://orcid.org/0000-0001-7619-9464

E.C. Vellinga https://orcid.org/0000-0003-4171-0554

N. Thongklang https://orcid.org/0000-0001-9337-5001

### Data availability

All of the data that support the findings of this study are available in the main text.
